# Home-Based mHealth Platform (Active-Feet) for Children With Idiopathic Toe Walking: Design, Development, and Acceptability Study

**DOI:** 10.2196/60867

**Published:** 2025-08-26

**Authors:** Miguel Membrilla-Mesa, Jose Heredia-Jiménez, Carla Di Caudo, Maria Almudena Serrano-Garcia, Yolanda Archilla Bonilla, Angel Ruiz-Zafra, Kawtar Benghazi-Akhlaki, Manuel Noguera-Garcia, Alberto Ortiz de Andres, Simon Perez-Garcia, Rocio Pozuelo-Calvo

**Affiliations:** 1 Department of Physical Medicine and Rehabilitation, Hospital Universitario Virgen de las Nieves, Granada, Spain; 2 Instituto de Investigación Biosanitaria ibs.GRANADA, Avda. de Madrid, 15, Granada, 18012, Spain, 34 958021574; 3 Department of Physical Education and Sports, University of Granada, Granada, Spain; 4 Centro Neuromédico, Granada, Spain; 5 Department of Radiology and Physical Medicine, University of Granada, Granada, Spain; 6 Department of Software Engineering, ETSIIT University of Granada, Granada, Spain

**Keywords:** digital health, mHealth, mobile health, avatar, platform, children and caregivers, paediatric, idiopathic toe walking, telerehabilitation, app

## Abstract

**Background:**

Physical exercise and stretching programs are the best initial options to treat idiopathic toe walking (ITW). These programs are designed to improve the flexibility and strength of lower limb muscles, enhancing the ankle’s range of motion and allowing for a normal gait pattern. In the pediatric population, one of the major limitations reported by therapists is low adherence to rehabilitation treatments or a lack of follow-up. In this context, children using mobile health (mHealth) tools could play an active and central role in their treatment of ITW, while mobile apps could also allow for daily monitoring by health care professionals.

**Objective:**

This study aims to design and develop a mHealth platform for individuals with ITW. In addition, a feasibility and acceptance test of a home-based exercise program was conducted using a comprehensive mobile app intended to improve walking in children with ITW.

**Methods:**

This study describes the context, content preparation, and mHealth platform design, as well as subsequent evaluation using a self-administered satisfaction questionnaire. Initially, the main features of the Active-Feet platform were discussed, focusing on its primary goal of helping children with ITW adhere to the rehabilitation program. However, this study did not evaluate the platform’s effectiveness in improving adherence or ankle range of motion. A set of 3D avatars consisting of animated characters was designed. Posterior muscle chain stretching exercises were selected following the main guidelines. The Active-Feet development process was carried out in 5 stages: requirements specification, platform design, platform implementation, platform deployment, and alpha testing of the app.

**Results:**

The final version of the Active-Feet app was evaluated from both the parents’ and children’s perspectives. Twenty patients and 1 parent per child assessed the app over 2 weeks and answered specially designed questionnaires. Parents rated the app’s impact on their child’s motivation and its overall effectiveness highly, with median scores of 4 (IQR 4-4). Notably, the item related to reconciling family life with rehabilitation treatment received a median score of 5 (IQR 4-5). Children’s responses also indicated positive ratings for motivation and user-friendliness, with a median score of 4 (very good; IQR 3.25-4). Questions about the app’s impact, helpfulness in learning, and exercise mirroring received a median score of 3 (good; IQR 3-4).

**Conclusion:**

This study describes the development process and testing of Active-Feet, an mHealth-based platform designed to offer treatment for children with ITW. After the long process, an attractive and easy-to-use platform for ITW was developed for the first time.

## Introduction

The normal gait pattern is acquired during growth. Toe-walking is generally regarded as the absence or limitation of heel strike during the initial contact phase of the gait cycle [1-3]. This condition is considered a common and normal deviation until the age of 3 years; however, to keep this gait pattern beyond that age could be pathological, and a specialist evaluation should be considered [4-7].

Toe-walking is a feature present in several neuromuscular conditions, such as cerebral palsy and muscular dystrophy, as well as behavioral disorders such as autism spectrum disorder [5]. However, it can be the result of a simple contraction or shortening of the lower limb muscles, specifically the hamstrings and triceps surae. Idiopathic toe walking (ITW) is a condition of unknown etiology. Currently, ITW is a diagnosis that can only be reached once other causes for a similar pattern of gait have been excluded, mainly cerebral palsy [8,9], muscular dystrophy [10], neuropathy [11], or foot malformation [12]. ITW represents a common referral to different specialists, and the prevalence was reported to be between 5% and 12% in the pediatric population, without gender differences [7].

A comprehensive assessment of children with ITW involves a detailed physical examination and observational gait analysis. This includes evaluating the range of motion (ROM) of lower limb joints, muscle strength, and performing muscle shortening tests, along with assessing the rotational profile of the lower limbs. This approach is essential to support a clinical diagnosis and to decide the proper and most effective individualized treatment for children with ITW. Electromyography, instrumented gait analysis [4,13], or the validated “Toe Walking Tool” can be used to support the presence of ITW [14,15].

The treatment options for ITW are based on age and the presence of limited ankle ROM due to muscle shortening. Nonsurgical treatments, stretching exercises, and orthotics are the main treatment options for children who have persistent toe walking with normal ankle ROM. However, if decreased ankle dorsiflexion flexibility exists, the treatment options are intended to improve the ankle ROM using serial casting, botulinum toxin, or surgery followed by orthosis to maintain the improvement and support gait training [16,17]. The 2019 Cochrane systematic review evaluated the effects of various interventions for ITW, including both conservative and surgical treatments. While the review aimed to assess improvements in ROM, it concluded that there were no significant differences in ROM between treatment groups due to the very low certainty of the evidence [18]. Physical exercise and stretching programs are designed to improve the flexibility and strength of lower limb muscles, improving the ankle ROM and reaching a normal gait pattern. Stretching exercises could contribute to maintaining the ROM obtained after surgical treatment [16]. Exercise and stretching programs can therefore be an effective and noninvasive option for the treatment of ITW.

In recent years, there has been a great expansion of health interventions mediated through new technologies. This has been called eHealth, and it includes the use of electronic technologies and digital communication to improve health care delivery. Mobile health (mHealth) interventions have emerged to facilitate and simplify access to medical care. They allow the collection of patient care–relevant data in real time and the use of this information to monitor, diagnose, and treat patients [19]. There are different mHealth interventions that contribute to the management of certain pathologies in pediatric rehabilitation programs, such as tools developed for parents and caregivers for children with cerebral palsy [17,20,21]. These apps should be developed under the participation, advice, and supervision of health care professionals to ensure the proper functioning for the benefit of both patients and health care systems [21].

In the pediatric population, loss to follow-up is common in children with ITW [22]. One of the primary challenges reported by therapists is low adherence to home exercise programs after the conclusion of physiotherapy sessions. This observation is supported by clinical practice experiences and discussions among specialists, underscoring the necessity for innovative tools such as the Active-Feet app to enhance adherence and support long-term therapeutic outcomes. There is a growing body of evidence supporting examples where physiotherapy has benefited from digital health technologies, primarily existing in the management of musculoskeletal [23] or neurological issues [24,25]. Specific exercise tools, virtual reality, and gaming have positively influenced rehabilitation [26]. Telerehabilitation focused on children with ITW is supported by the concept that this population has grown up in a technological environment and that mobile devices are a common way for them to interact with others.

A mHealth platform specially designed to meet the needs of children with ITW would be an innovative approach to improve both adherence and effectiveness of rehabilitation programs. Furthermore, children using mHealth tools could play an active and central role in treatment and, at the same time, the mobile app would allow daily monitoring of treatment by the health care professional.

This study aims to develop and evaluate an mHealth-based platform for children with ITW. In addition, it tested the feasibility and acceptance of a home-based exercise program using a comprehensive mHealth tool to improve walking in patients with ITW. This study describes the context study, content preparation, and mobile app design, with subsequent evaluation using a self-administered satisfaction questionnaire from the perspective of parents and children.

## Methods

### Study Design Overview

The reference population for the content development was children aged between 7 to 12 years with a diagnosis of idiopathic toe walking who attended a pediatric rehabilitation outpatient clinic. This study was conducted in three phases: (1) assessment of children with ITW needs for a home-based rehabilitation program (content preparation phase), (2) designing an attractive mobile app for children (app design phase), and (3) evaluating the app from the user’s perspective (app evaluation phase).

**Figure 1.**
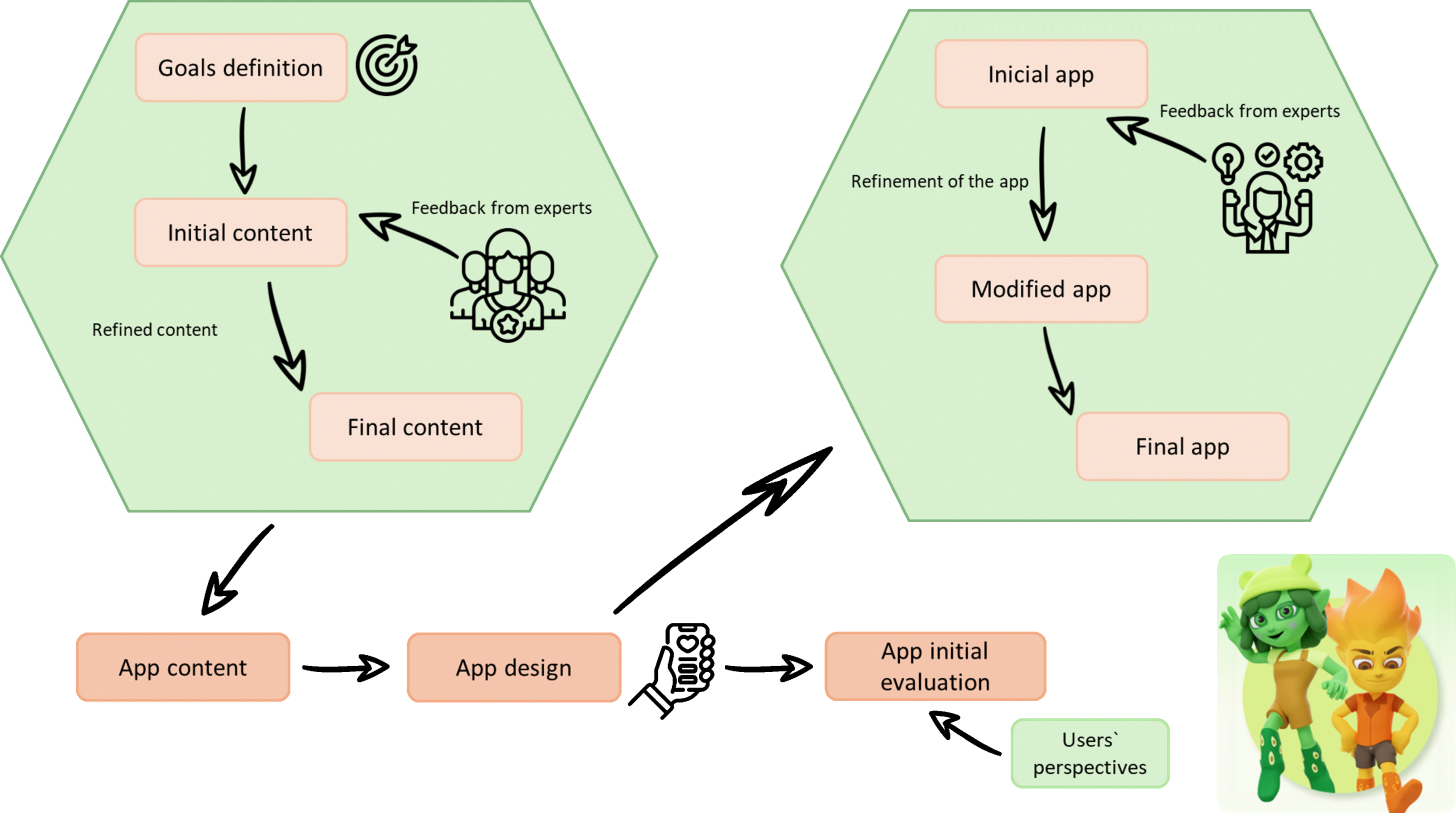
A schematic view of the main steps followed in the development of the Active-Feet app.

The development process, from conceptualization to pilot testing of the Active-Feet app, is outlined in Figure 1.

### Content Preparation Phase

The original idea of developing a specific app for home-based exercise treatment in children with ITW arose from the monthly multidisciplinary meetings that take place in the pediatric outpatient rehabilitation clinic. A physiatrist, physiotherapist, and occupational therapist were involved in the initial content preparation. There were several reasons to consider a mHealth approach for rehabilitation in ITW, some of which are as follows: (1) an increasing number of referrals of children with ITW for physiotherapy treatment, (2) low post-treatment compliance with home-based exercise programs, despite written instructions on their execution. This observation is supported by clinical practice experiences and discussions among specialists, highlighting the need for tools such as an app to improve adherence, (3) the number of trips to the hospital for children and caregivers becomes disruptive for daily routines, so continuing physiotherapy sessions at the hospital was not an option for many families, and (4) performing repetitive exercises at home is not attractive for many children and could become a problem in the relationship between parents and children.

An exploratory search was carried out by researchers in PubMed, the Apple App Store, and Google Play Store, and no mobile app related to the treatment of children with ITW was found.

Initially, the main features of the Active-Feet platform were discussed, focusing on its primary goal of helping children with ITW adhere to the rehabilitation program. The app should allow health care professionals to view and to track children’s compliance and execution of the exercises. At the same time, the patients could visualize themselves performing the exercises using the device’s own camera. It was also considered important to develop a suitable and visually attractive app design for the pediatric population. The app should be intuitive, easy to use, and simple to access to avoid disuse. In addition, the app should be a communication channel between the physician or therapist and the patient.

**Figure 2.**
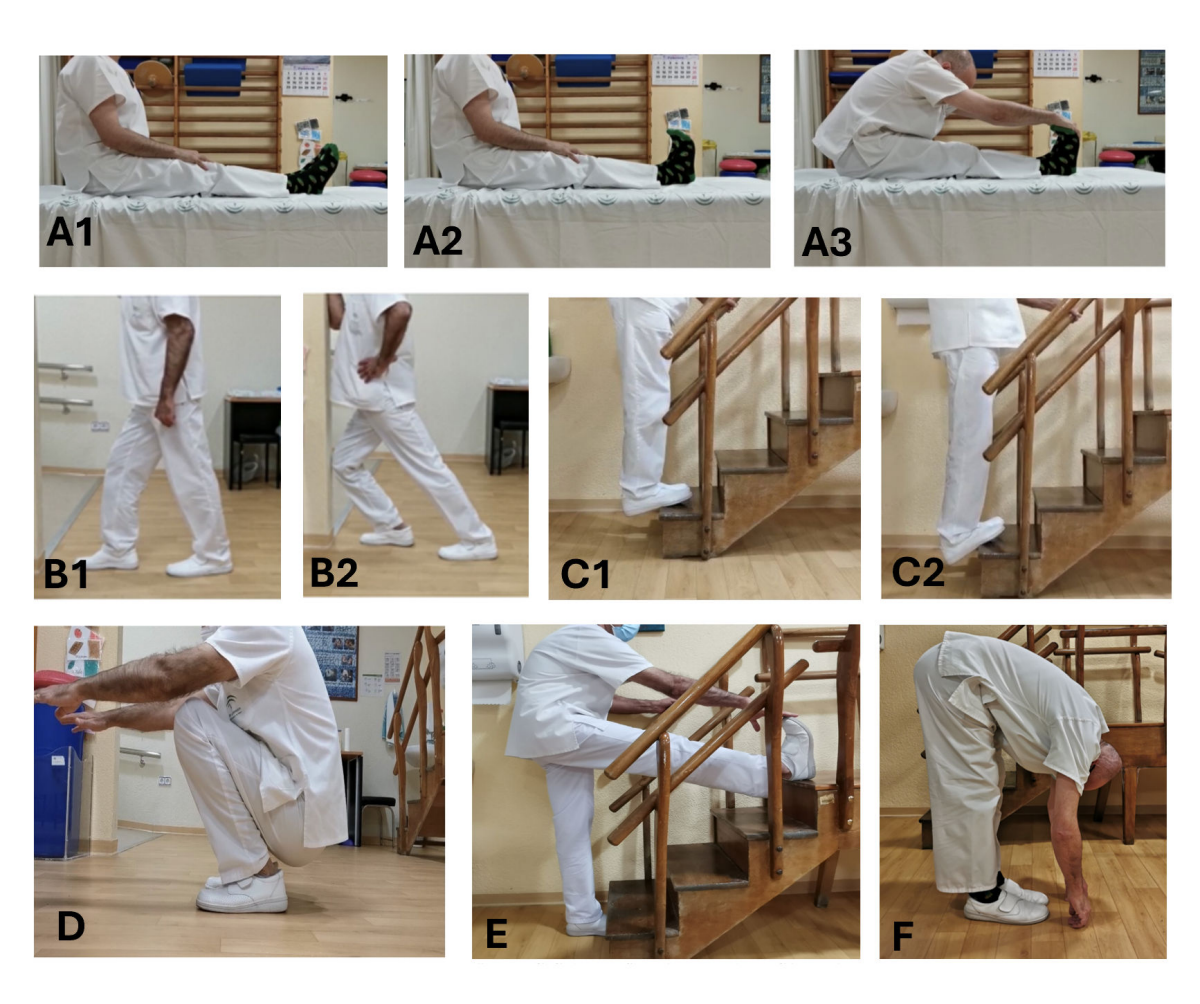
Main exercises: (A1-A3) posterior muscle chain sitting stretch, (B1-B2) triceps surae stretch, (C1-C2) stair (ladder) stretch, (D) soleus stretch, (E) one-leg hamstring stretch, and (F) posterior muscle chain standing stretch.

In successive meetings of the multidisciplinary team, a consensus was reached regarding the type of exercises recommended for ITW. After reviewing different guidelines and considering that muscle shortening is a frequent finding, posterior muscle chain stretching exercises were chosen. The selected exercises are presented in Figure 2 and include posterior muscle chain stretches while sitting (A1-A3) and standing (F), triceps surae stretches standing (B1-B2) and using stairs (C1-C2), stretching the soleus (D), and one-leg hamstring stretch. Finally, the initial content was evaluated by senior specialists (1 physiatrist, 1 physiotherapist, and 1 occupational therapist) to get expert opinions. The initial content was finalized after applying the experts’ recommendations.

The first meeting with the computer scientists involved in the creation of the app was established. It was decided that the app should include three user profiles: administrator, patient, and health care professional. The health care professional profile (physician or therapist) should include functionality to design a customized exercise protocol for each patient. The health care professional could increase or decrease the number or intensity of sessions, eliminating or changing some of the proposed exercises and reviewing the sessions already carried out. In the same way, communications between children and therapists could be performed using the chat included in the app. The administrator role is fulfilled by both the physician, who registers the child in the app, and the therapist, who selects and adjusts the exercises weekly based on the patient’s progress, including the number of sessions completed and the time dedicated to the rehabilitation program. This collaborative approach ensures both medical oversight and personalized therapeutic guidance.

Once the first design was reviewed, the researchers concluded that the appearance of the app was unattractive for children, so it was suggested to design 3D animated avatars to demonstrate the exercises as tutors. It was agreed that the avatars should meet certain requirements. The proposed characters should be visually attractive; they should bring the attention to the feet, the most important part of the body in this case. Therefore, the feet should be well-defined and subtly larger to highlight their relevance, barefoot to better visualize their movement, but they should wear striking socks to add extra visual appeal to the design.

**Figure 3.**
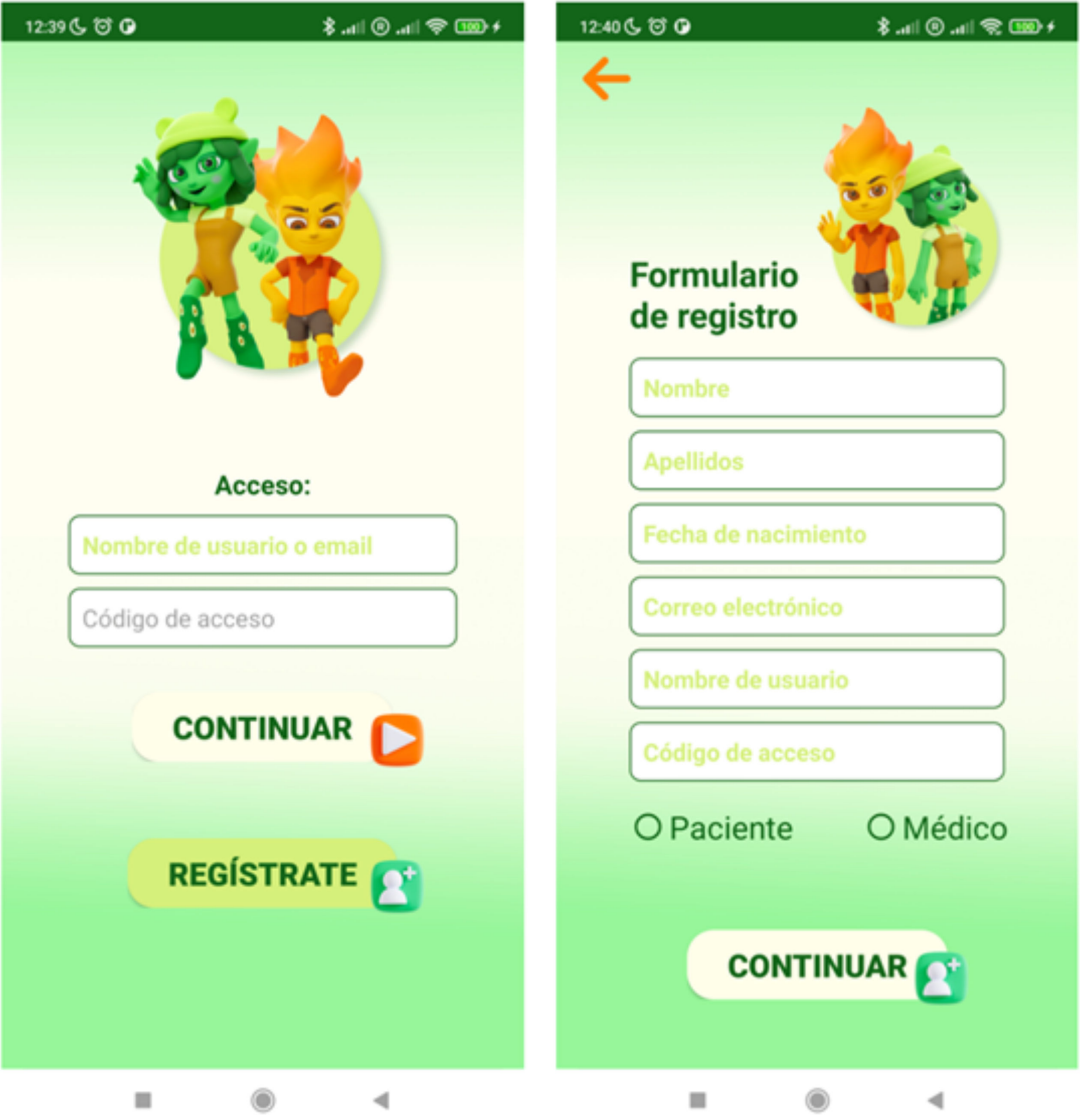
Screenshots from the Active-Feet app showing the characters: TOBI and TOE. View to log in (left) and to request registration by a new user (right).

From this entire process, TOBI and TOE characters emerged, 3D avatars enabling the guiding of the patient with ITW in performing the exercises were designed (Figure 3). At this point, the physiotherapists specialized in carrying out the chosen stretching exercises recorded themselves, and the recordings were used as templates for the avatars’ motion. The exercises performed by the 3D avatars were designed to be simple and at the same time easy to mirror by children.

### Platform Development Phase

The platform development process was carried out over five stages, executed sequentially: (1) requirements specification, (2) platform design, (3) platform implementation, (4) platform deployment and alpha testing of the mobile app, and (5) validation and performance testing of the mobile app (beta phase).

#### Stage 1: Requirements Specification

Tasks were conducted to gather both functional and nonfunctional requirements, aiming to specify the system’s characteristics in terms of functionality (functional requirements): types of users, types of rehabilitation sessions, how interactions between patients and doctors would occur, and other details. In addition, nonfunctional requirements were addressed, primarily tackling three issues: (1) deployment architecture, (2) the need to enhance usability and ensure the accessibility of the app due to the needs of the end users (young patients), and (3) data protection.

#### Stage 2: Platform Design

In accordance with the requirements (functional and nonfunctional), a design phase was carried out. As a result of this phase, a list of use cases enumerating all the functionalities that should be present in the platform was obtained.

**Figure 4.**
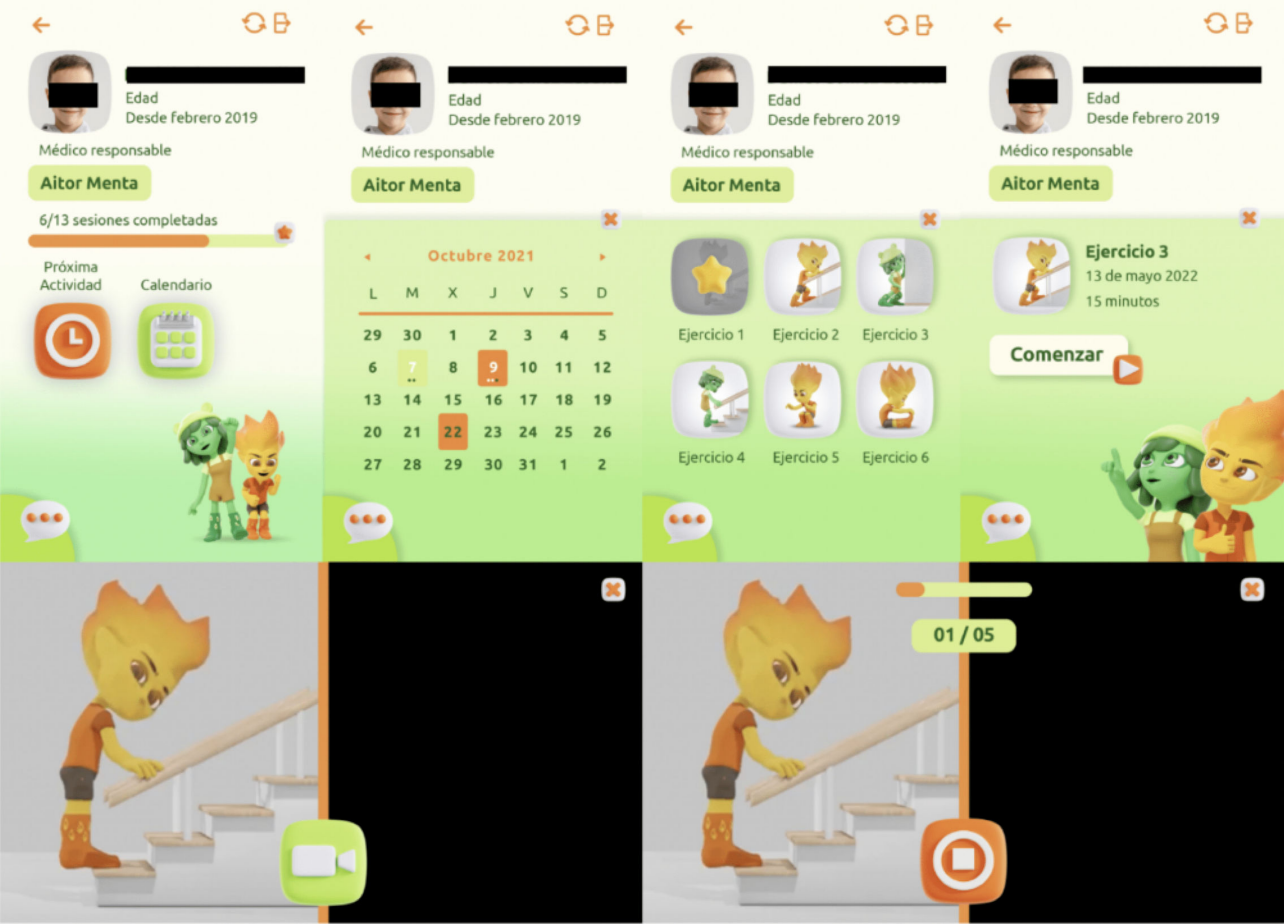
Sample of screenshots from the Active-Feet app patient profile.

Furthermore, as a result of designing according to the nonfunctional requirements, it was agreed to use a client-server architecture with a single multiuser mobile app (for patients and doctors) that would display different views depending on the role, as well as another view for the system administrator, which has typical administrative functions. (Figures4  5 and Multimedia Appendix 1). The server is planned to serve as a provider of web services to implement part of the functionalities and as a database for information management and storage.

**Figure 5.**
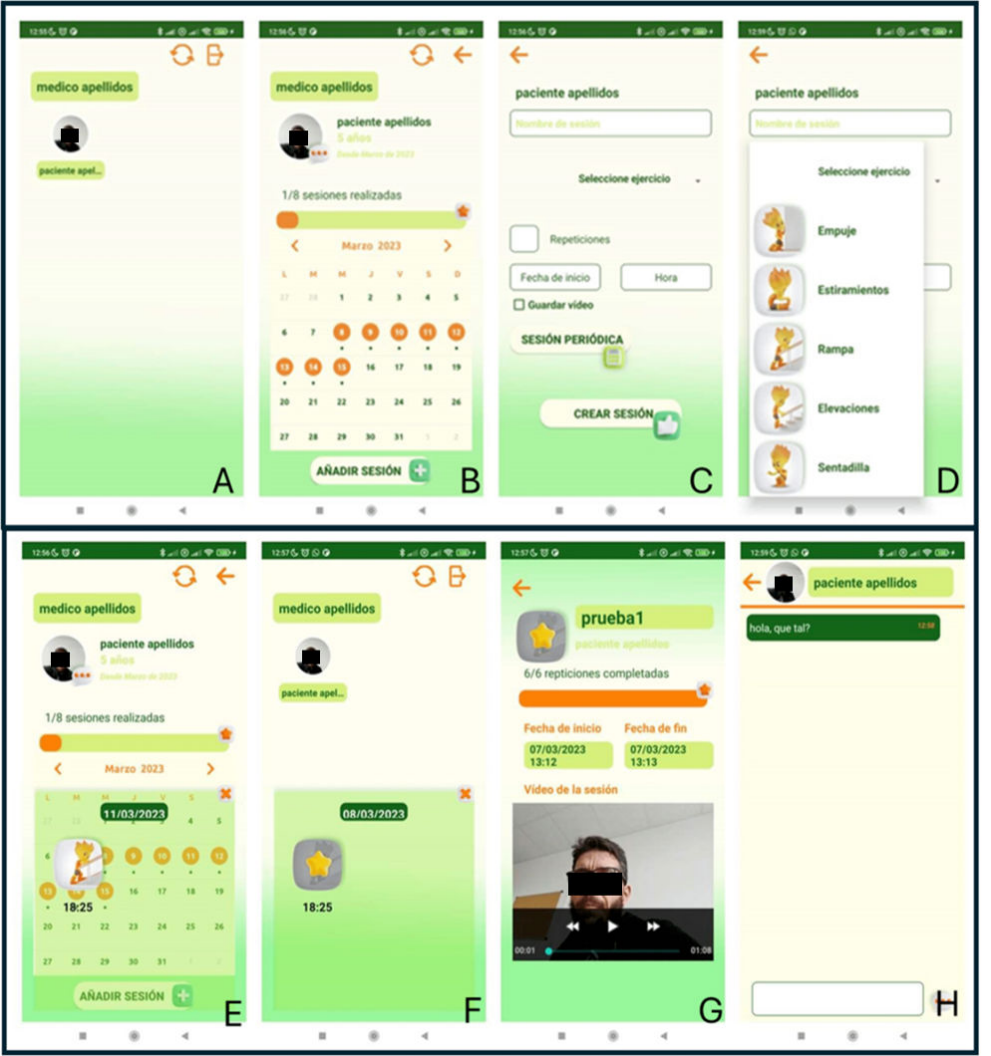
Sample of screenshots from Active-Feet app therapist or physician profile. (**A**) List of patients assigned, (**B**) summary of scheduled session from patient profile, (**C**) view to create a new session, (**D**) list of available exercises, (**E**) exercise scheduled to do, (**F**) exercise already done, (**G**) exercise review from record, (**H**) view from personal chat with the patient.

To ensure usability, the services of a company specializing in mobile app interface design were used. This company provided wireframes for the mobile app designed with a user-centered approach, aiming for maximum usability and accessibility.

The Active-Feet platform comprises two deployment environments: a secure backend hosted on a private server and a mobile app. The backend operates on a virtual machine with robust security and reliability measures. The mobile app was tested on 4 physical devices (2 smartphones and 2 tablets) and 2 virtual devices. Physical devices, such as the Xiaomi Redmi Note 12 and Samsung Galaxy Tab A8, demonstrated superior performance due to their advanced hardware. Conversely, the Samsung Galaxy Tab A7 Lite exhibited the lowest performance, likely due to its limited RAM and internal processes. Virtual devices also performed adequately, showcasing the platform’s adaptability across varying specifications. (More detailed information is included in Multimedia Appendix 2). Overall, the platform proved compatible with low-to-mid-range devices, ensuring broad accessibility and delivering a satisfactory user experience, particularly on devices with greater processing power.

Finally, to ensure access to information, protocols were established so that only a patient and their associated doctor could access the patient’s data. Similarly, doctors cannot view other doctors or patients for whom they are not responsible. Only the administrator can view all information. To achieve this, access control mechanisms were implemented. Furthermore, to ensure secure information exchange, all requests between the client (mobile app) and server are made securely using HTTPS and JSON Web Token.

#### Stage 3: Platform Implementation

The platform consists of blocks: the server-side (backend) and the mobile app (frontend). The server-side (backend) was developed using the Python programming language (Python Software Foundation) and the Flask framework (Pallet Projects) for web service creation. MongoDB (Mongo Inc.) has been used as the database management system. On the other hand, the mobile app (frontend) was implemented following the wireframes provided by the design company and implemented for the Android (Google LLC) operating system, compatible with versions 5.0 and later (since 2014).

#### Stage 4: Platform Deployment and Mobile App Alpha Testing

The backend (server) was deployed on a private server belonging to the University of Granada network, which was publicly accessible and equipped with security certificates. The mobile app was tested on three different devices: Xiaomi Redmi Note 8, Xiaomi Redmi Note 9, and Xiaomi Redmi Note 10. Validation tests were conducted to assess functionalities, and common errors derived from the implementation were corrected.

#### Stage 5: Validation and Mobile App Performance Testing (Beta Phase)

This stage is currently under development. The evaluation of the direct impact on clinical outcomes is being conducted as a second phase of the study by the research group. These follow-up investigations aim to evaluate the app’s effectiveness in achieving functional improvements in children with ITW. The evaluation of exercise accuracy using the Active-Feet app will be carried out by the therapist through visual monitoring during each session. The therapist would observe the patient’s posture to ensure proper alignment and movement, focusing on potential compensations at the pelvis, hips, or knees. In addition, the therapist will check whether the patient dedicates the correct amount of time to each stretch, typically 15 seconds, as specified in the treatment protocol. This process ensures that exercises are performed correctly and safely.

Exercise intensity will be monitored by progressively increasing the number of weekly repetitions for each exercise. Since patients may experience discomfort due to muscle shortening at the start of the intervention, the progression will be carefully adjusted to allow gradual adaptation and avoid excessive strain. Similarly, frequency measurements will include tracking the number of patient connections to the app, the number of days the patient engages in sessions, and the duration of each session. These combined metrics aim to provide a comprehensive understanding of patient adherence, engagement, and rehabilitation progress.

### Evaluation of the App From the Users’ Perspective (App Evaluation Phase)

The final version of Active-Feet app was assessed considering the perspectives of parents and children on the usefulness and attractiveness of the app, as well as the ability to encourage children to follow the home-based exercise program.

Participants for this feasibility and acceptance pilot study were required to meet the following inclusion criteria: diagnosis of ITW, aged between 7- 12 years, and ability to use an Android smartphone (with appropriate parental supervision and support). Twenty selected participants who were available and willing to cooperate assessed the app for 2 weeks. To facilitate the use of the app, a first guided use of the Active-Feet app was carried out face-to-face in the outpatient clinic by a therapist, with a detailed explanation provided to the children and parents.

Specific questionnaires were designed by the researchers (refer to Multimedia Appendix 3) and administered to both parents and children following the 2-week app testing period. The answers from adults were measured based on a 5-point Likert scale, while the questionnaire for the pediatric population was evaluated using a 4-point visual analog scale. To summarize data distributions, the median and IQR (25th-75th percentile) were calculated for all Likert scale responses. After collecting the questionnaires from parents and children, the data were analyzed using SPSS software (version 24; IBM Corp).

### Ethical Considerations

Ethical approval for this study was obtained from the Granada Research Ethics Committee (CEI Granada; approval code: 95133). Written informed consent was obtained from all adult participants and from the parents or legal guardians of the children involved in the app evaluation. Children also provided assent prior to participation. All personal data collected during the study were anonymized to ensure participant privacy and confidentiality in accordance with applicable data protection regulations. No monetary compensation was provided to participants for their involvement in the study.

## Results

### Demographic and Physical Characteristics of the Participants

A total of 20 children participated in the study. The median age of the children was 10 (IQR 9-11) years, the median weight was 42.8 (IQR 35.6‐55.7) kg, and the median height was 148 (IQR 138‐160) cm.

Regarding ROM and flexibility measurements, the median plantar flexion was 70° for both the right and left sides, with IQRs of 60°‐75.5° for the right and 60°‐74° for the left. Median dorsiflexion with the knee extended was 0° on the right side (IQR −3.5° to 0°) and 1° on the left side (IQR −5° to 4°). With the knee flexed, median dorsiflexion was 10° bilaterally, with IQRs of 6°‐12° for the right and 8.5°‐13.5° for the left. The Silfverskiöld test was positive in all participants, confirming restricted dorsiflexion with the knee extended. Further details of the measurements conducted on the participants can be found in Multimedia Appendix 4.

### Characteristics of Parental and Children Questionnaires

Three main sections were considered in each questionnaire: (1) questions about the ease of use or user-friendliness, (2) questions about the impact of the app, and (3) attractiveness of the app and user feedback. All these aspects were addressed in both questionnaires using appropriate language for each group—parents and children.

A total of 20 parents, 1 per child in the study, completed the questionnaire. The ease of the registration process, learning to run the app, and access to different sections were the main questions assessed from parents (questions 1, 2, and 3). Regarding the impact of using the app, a question was addressed to the enhancement of their child’s motivation with the app (question 4). Finally, acceptance and attractiveness were addressed by asking whether the content was appropriate for their children (question 5) and whether the app was helpful for the reconciliation of family life and the rehabilitation process (question 6).

In the questionnaire addressed to the children (n=20), questions were asked about how much fun they found (question 1) and their motivation to do the exercises daily (question 5). They were also asked whether they liked the avatars, as a measure of acceptance and attractiveness (question 2). Regarding the impact of using the app, questions addressed how the avatars helped them do the exercises (question 3) and whether the exercises shown by the avatars were easy to mirror (question 4).

### Questionnaire Results From Parents and Children

The interaction between children and parents with the app technology was well received by all participants. In general, they were pleased with the quality of the graphical user interface, ease of navigation, and the overall impression of how patients interact with the mHealth system.

The parental question regarding the improvement of the child’s motivation to perform the exercises (question 4) received one of the highest scores, followed by the section addressing the impact of using the app (question 5). Finally, the aspects related to the app’s ease of use (questions 1 and 3) and learnability (question 2) were also rated highly by participants (Multimedia Appendix 5). The median score for the first 5 questions was 4 (IQR 4-4). The item referring to the reconciliation of family life with the rehabilitation treatment of their children (question 6) was highly appreciated by parents, showing a median of 5 (IQR 4-5). The overall mean percentage of parents responding “4” across all parental questions was 72.5%. Only 5% of the parents’ responses scored 3 or lower on the Likert scale.

Children’s responses to questions on user-friendliness and motivation demonstrated positive outcomes. For question 1, which assessed whether performing the exercises was an enjoyable experience, the median score was 4 (very good) with an IQR of 3.25-4. Similarly, for question 5, which evaluated whether children remembered to perform the exercises daily with TOBI and TOE, the median score was also 4, with an IQR of 4-4. Questions regarding the impact of use, the app’s helpfulness in learning (question 3), and the ease of mirroring exercises (question 4) received a median score of 3 (good) with an IQR of 3-4. Regarding the acceptance and attractiveness of the app (question 2), children expressed positive feedback, with a median score of 3 (IQR 3-4). They found the avatars to be delightful and engaging. The overall mean percentage of participants responding with a score of 4 across all children’s questions was 52%. No responses of “Bad” or “Very Bad” were recorded.

## Discussion

### Principal Findings

The aim of this study was to develop and implement an app for telerehabilitation in children with ITW. The findings of this research suggest that the Active-Feet mHealth-based platform is feasible for children. An attractive and easy-to-use app, designed to help patients perform exercises following the instructions of 3D avatars, was developed during the process. At the same time, the Active-Feet app allows for recording completed exercises, enabling the therapist to track the frequency, intensity, and accuracy of performance through their app profile. Corrections or recommendations via chat between the therapist and patient can improve interaction quality and provide guidance to users in the event of errors. Collecting real-time health care data from patients and using this information to monitor, diagnose, and treat them is one of the main advantages of mHealth tools [19].

The approach of ITW shows a lack of consensus regarding optimal conservative management. A wide variation in treatment across providers and institutions exists. However, stretching exercises are advocated as the core management for ITW [16,18]. Mobile apps can incorporate positive behavior change techniques to improve exercise adherence and thus optimize clinical benefits. In children with ITW, loss to follow-up is common [22], and long-term adherence remains challenging. Promoting the reconciliation of daily routines and rehabilitation treatment is important to avoid noncompliance to the treatment. Moreover, telerehabilitation improves patients’ access to rehabilitation services and saves patient time traveling to the hospital or rehabilitation center. Evidence suggests that there are many potential applications to be explored to enhance connectivity of the interdisciplinary rehabilitation team in the pediatric population [27]. All these elements were considered in developing the Active-Feet platform.

Although the evaluation of the Active-Feet app did not use the Mobile App Rating Scale (MARS) used in previous studies, the questionnaires administered to assess the app revealed higher user engagement and satisfaction scores compared to similar mHealth apps. For example, pain-related apps evaluated with MARS achieved an average overall score of 3.17 out of 5, with functionality rated the highest and engagement often scoring lower [28]. In contrast, the Active-Feet app demonstrated strong user satisfaction and engagement among parents and children, highlighting its ability to effectively meet the needs of its target population. These findings emphasize the potential of the Active-Feet app to serve as a valuable tool for enhancing adherence and motivation in rehabilitation contexts.

### App Usability and Acceptability

This study describes the entire process of developing a specific app to perform the rehabilitation treatment for ITW in a complex population. The Active-Feet app showed to be attractive and easy enough to use for children and parents. Currently, there is no other app that has been developed for this condition, so no comparison can be made. The reference for this research was supported and guided by previous home rehabilitation interventions administered from our group of researchers in developing mHealth systems [29,30]. This paper assembles the preliminary qualitative and quantitative data on usability and acceptability of the Active-Feet app for conducting the home-based exercise program and to explore potential outcomes of the intervention. The direct impact on clinical outcomes such as adherence to rehabilitation protocols or improvements in ROM was not assessed in this study. Future research should focus on overcoming these limitations by using longitudinal studies with objective measures to assess how effective the app is in improving adherence and functional outcomes in children with ITW.

### Potential Broader Applications

It is important to consider the impact on special groups of children using digital characters or avatars during rehabilitation treatment and the advantages of these kinds of mHealth systems. It was reported that patients with autism have shown benefits, such as being able to resemble the movements of the avatar [31]; improved the ability to recognize and express basic emotions [32]; and increased the capability for social interactions by practicing verbal and nonverbal behavior in virtual reality environments [33]. Autistic children who exhibit toe walking are generally not classified as ITW, and additional considerations may be needed for their cognitive and behavioral differences. Consequently, while this app is primarily designed for idiopathic toe walkers, it could also serve as a valuable tool for children with toe-walking behaviors associated with conditions such as autism or other motor impairments, provided appropriate adaptations are made. In addition, a recent systematic review of mobile apps for patients with chronic conditions or multimorbidity has called for further research to develop and evaluate apps that are both high quality and have a high capacity to promote positive behavior change in patients [34].

### Limitations

This study had additional limitations. Twenty participants collaborated with the researchers to assess the app. Although some studies suggest that this sample size in usability tests can identify over 80% of usability issues [35,36], it may not capture all potential problems. In future research, we should consider a larger sample size and a more extended period of follow-up. The missing information regarding the participants’ previous knowledge of the exercises was another limitation of this study. In addition, the assessment of exercise accuracy relied on visual monitoring by the therapist; however, the lack of quantitative metrics such as joint movement tracking may limit the precision and consistency of exercise evaluation. Future research could benefit from incorporating motion tracking technologies to provide more objective and accurate assessments of exercise performance.

The use of a custom-designed satisfaction questionnaire instead of a standardized usability assessment tool, such as the System Usability Scale (SUS), was also a constraint in our research. While our questionnaire was specifically tailored to suit the linguistic and cognitive needs of a pediatric population, it limits the comparability of our findings with those of other studies. Future studies should consider incorporating validated and widely used scales to enhance the generalizability of results.

### Strengths and Future Directions

Finally, the findings of this study reveal the structure and process aspects of developing and optimizing a mobile app aimed at the pediatric population. However, the implementation of the Active-Feet app is not recommended until feasibility and effectiveness have been demonstrated in appropriately designed randomized controlled trials. This study also has strengths. First, the acceptance and satisfaction of the intervention have been demonstrated by users and parents. Second, the Active-Feet app was developed for both mobile and tablet devices. Third, to our knowledge, this is the first study showing the entire process of a mHealth platform created to deliver telerehabilitation programs for children with ITW.

### Conclusions

Exercise and stretching programs can be an effective, noninvasive option for the treatment of ITW in children, and the use of mobile apps is an alternative for home-based treatment programs. Although the Active-Feet app has been implemented in patients with ITW, its use could potentially be extended to children who exhibit toe walking as a frequent finding, such as those with autism spectrum disorders. This study describes the entire process of developing a mHealth system, and an attractive, easy-to-use mHealth platform for children with ITW was developed for the first time. Future studies should evaluate long-term effectiveness of the app and determine whether it should be recommended for patients, relatives, or health care professionals.

## Supplementary material

10.2196/60867 Multimedia Appendix 1 Sample of screenshots from Active-Feet app administrator profile.

10.2196/60867 Multimedia Appendix 2 Technical specifications physical devices.

10.2196/60867 Multimedia Appendix 3 Questionnaire for parents and children.

10.2196/60867 Multimedia Appendix 4 Demographic and physical characteristics of the participants.

10.2196/60867 Multimedia Appendix 5 Results from questionnaires with the Active-Feet app performed by parents and children. (A) Frequencies of parents’ responses and (B) frequencies of children’s responses.

## Abbreviations

ITW: idiopathic toe walking
MARS: Mobile App Rating Scale
mHealth: mobile health
ROM: range of motion
SUS: System Usability Scale
