# Supplementary figures and images for: Home-Based mHealth Platform (Active-Feet) for Children With Idiopathic Toe Walking: Design, Development, and Acceptability Study
Source: JMIR Rehabil Assist Technol. 2025 Aug 26;12:e60867. doi: 10.2196/60867 (PMC12380407; doi:10.2196/60867)

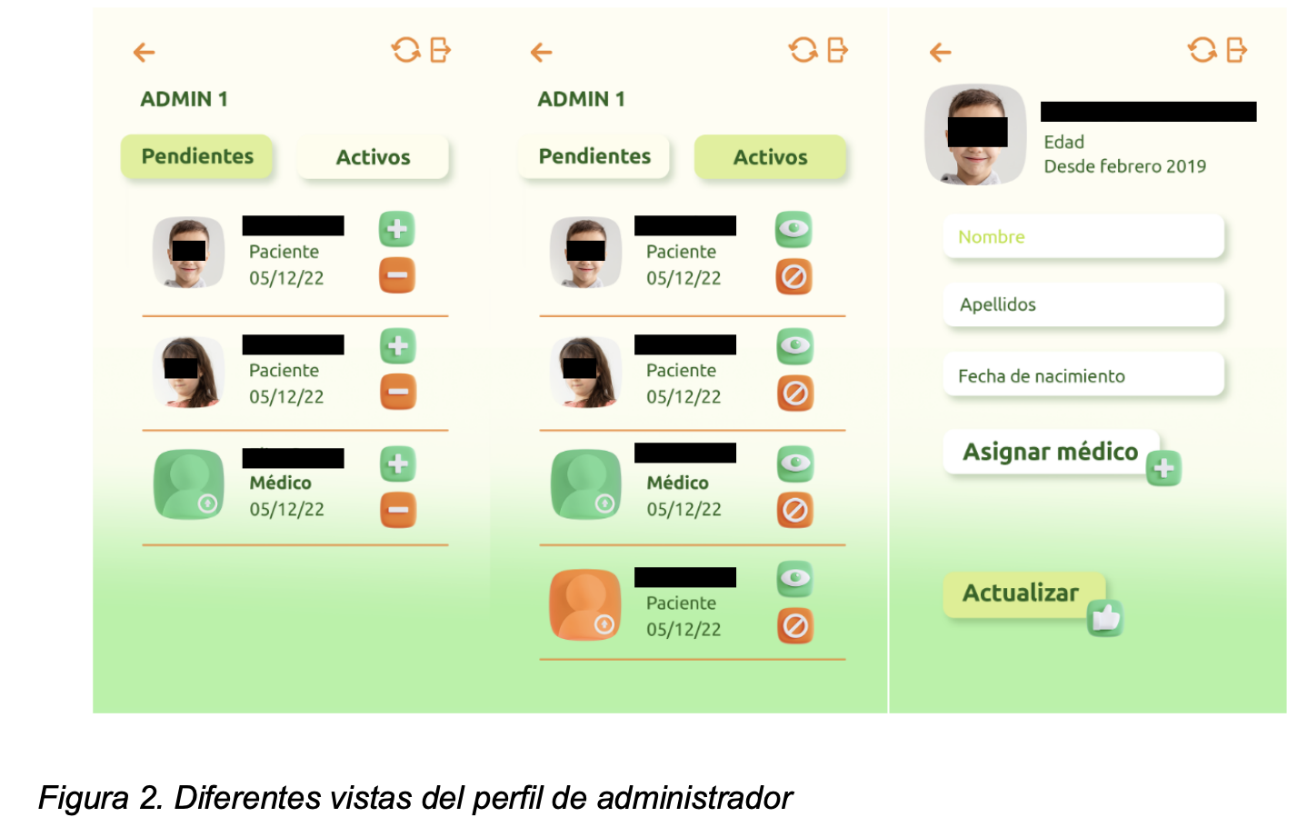

Supplement: Multimedia Appendix 1 [file rehab-v12-e60867-s001.docx]

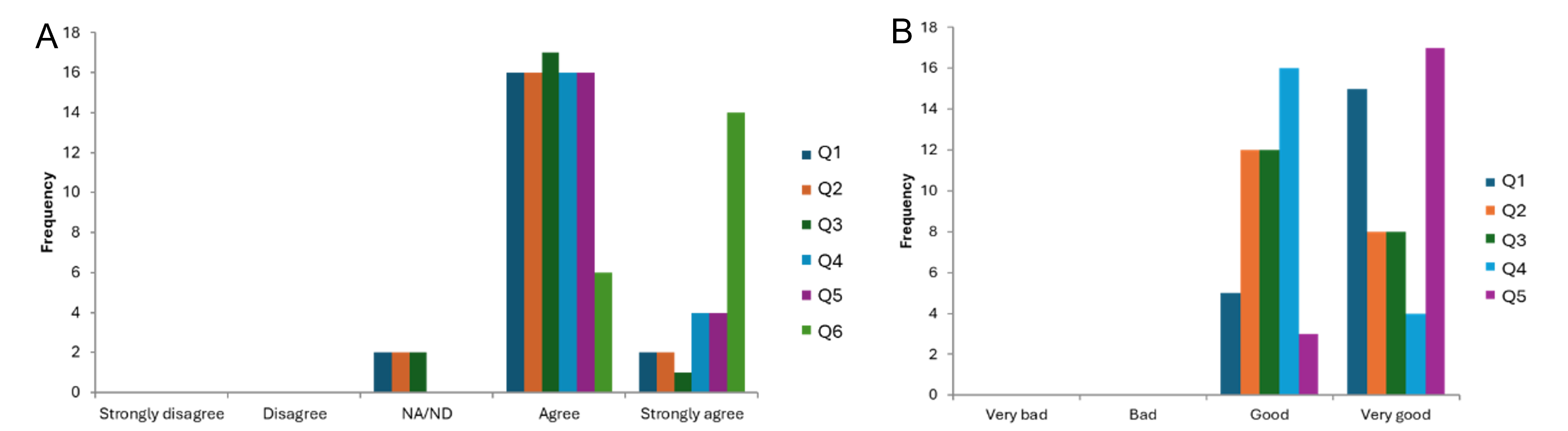

Supplement: Multimedia Appendix 5 [file rehab-v12-e60867-s005.png]
