# Supplementary material for: Home-Based mHealth Platform (Active-Feet) for Children With Idiopathic Toe Walking: Design, Development, and Acceptability Study
Source: JMIR Rehabil Assist Technol. 2025 Aug 26;12:e60867. doi: 10.2196/60867 (PMC12380407; doi:10.2196/60867)
Supplement: Multimedia Appendix 2 [file rehab-v12-e60867-s002.docx]

| Feature | Galaxy Tab A7 Lite | Galaxy Tab A8 | Xiaomi Redmi Note 8 | Xiaomi Redmi Note 12 |
| --- | --- | --- | --- | --- |
| Display | 8.7" TFT, 1340x800 px | 10.5" TFT, 1920x1200 px | 6.3" IPS LCD, 2340x1080 px | 6.67" AMOLED, 2400x1080 px |
| Processor | MediaTek Helio P22T | Unisoc Tiger T618 | Qualcomm Snapdragon 665 | Qualcomm Snapdragon 685 |
| RAM | 3 GB | 4 GB | 4 GB | 8 GB |
| Storage | 32 GB (expandable) | 128 GB (expandable) | 128 GB (expandable) | 256 GB (expandable) |
| Rear Camera | 8 MP | 8 MP | 48 MP + 8 MP + 2 MP + 2 MP | 50 MP + 8 MP + 2 MP |
| Front Camera | 2 MP | 5 MP | 13 MP | 13 MP |
| Battery | 5100 mAh, 15W fast charge | 7040 mAh, 15W fast charge | 4000 mAh, 18W fast charge | 5000 mAh, 33W fast charge |
| Operating System | Android 11 (One UI) | Android 12 | Android 9 (MIUI, upgradable) | Android 13 (MIUI 14) |
| Weight | 366-371 g | 508 g | 190 g | 183.5 g |
| Connectivity | Wi-Fi / LTE | Wi-Fi / LTE | 4G LTE | 4G LTE |
| Extras | Metallic body | Dolby Atmos speakers | Gorilla Glass 5 | AMOLED display, 120 Hz |
| Approximate Price | $120-$150 USD | $180-$250 USD | $150-$200 USD | $200-$300 USD |
