# Supplementary material for: Home-Based mHealth Platform (Active-Feet) for Children With Idiopathic Toe Walking: Design, Development, and Acceptability Study
Source: JMIR Rehabil Assist Technol. 2025 Aug 26;12:e60867. doi: 10.2196/60867 (PMC12380407; doi:10.2196/60867)
Supplement: Multimedia Appendix 3 [file rehab-v12-e60867-s003.docx]

**Satisfaction Questionnaire for Parents**

**1. To enter to the app was easy.**

1 2 3 4 5

Strongly Disagree Disagree Neutral Agree Strongly Agree

(Very difficult or improper) (Very easy or proper)

**2. To learn to use the app was easy.**

1 2 3 4 5

**3. Accessing the different sections of the application was easy.**

1 2 3 4 5

**4. Active-Feet app improves the motivation of the kid to do the exercises**

1 2 3 4 5

**5. The content of the app was appropriate for the child age.**

1 2 3 4 5

**6. The use of the app allowed to reconcile the family life and rehabilitation process.**

1 2 3 4 5

**Satisfaction Questionnaires for patients**

**1. To do the exercises was a good time.**


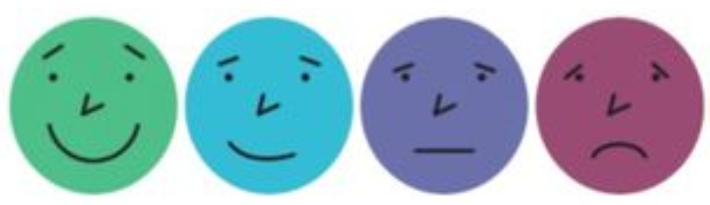

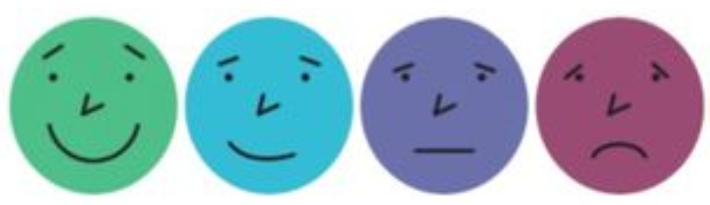

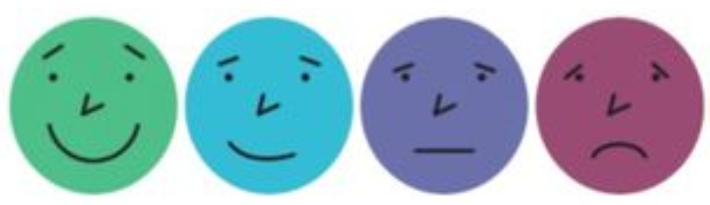

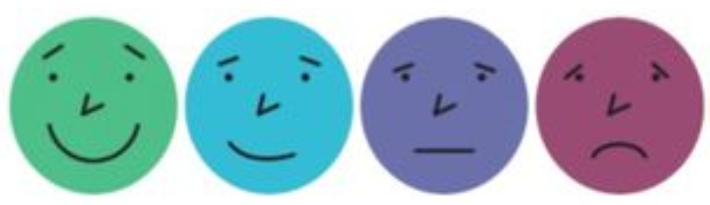


Very bad Bad Good Very Good

**2. I like Tobi and Toe, they are nice.**


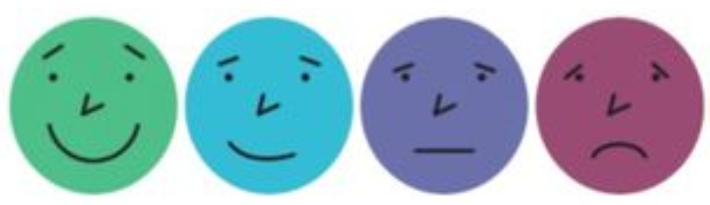

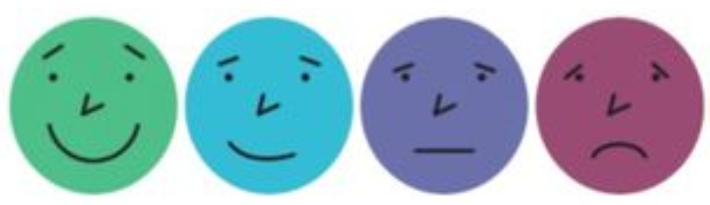

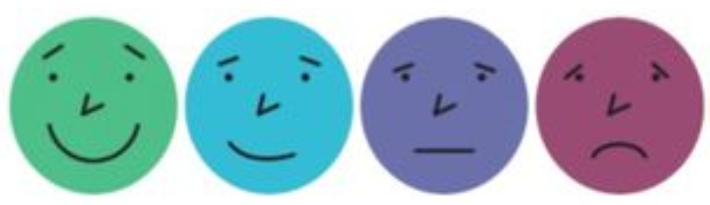

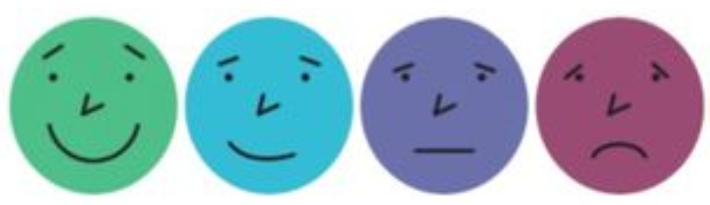


Very bad Bad Good Very Good

**3. Tobi/Toe help me to learn how to do the exercises.**


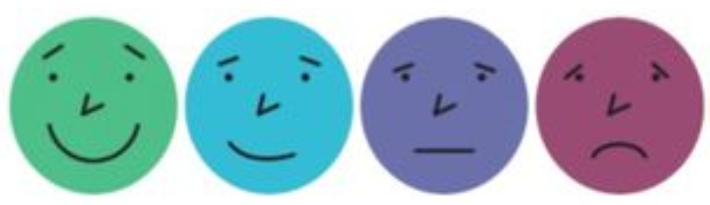

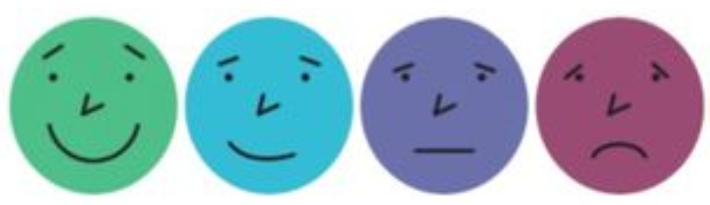

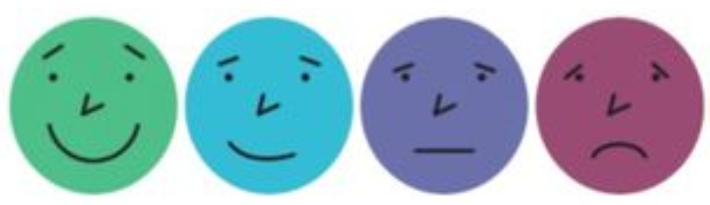

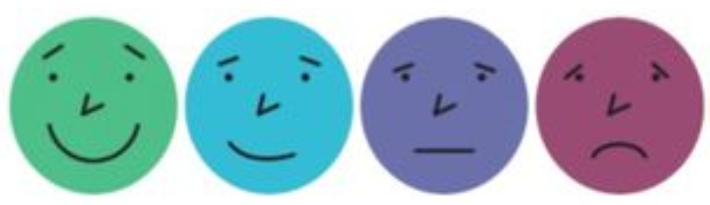


Very bad Bad Good Very Good

**4. The exercises are easy.**


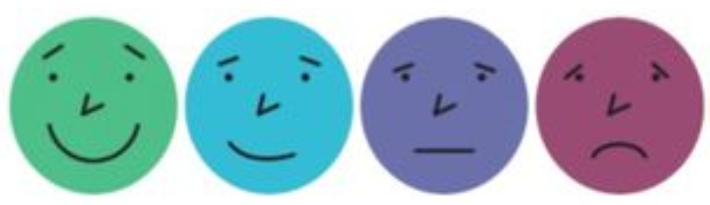

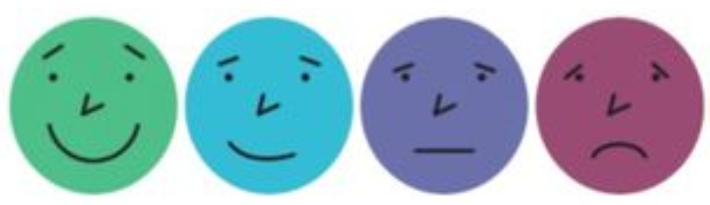

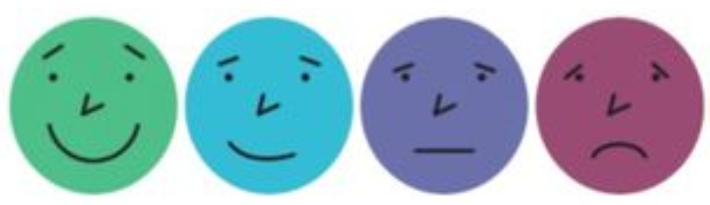

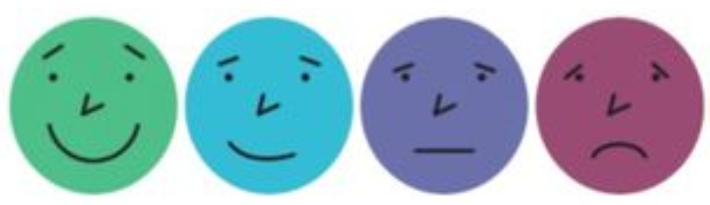


Very bad Bad Good Very Good

**5. I remember to do the exercises everyday with Tobi and Toe**


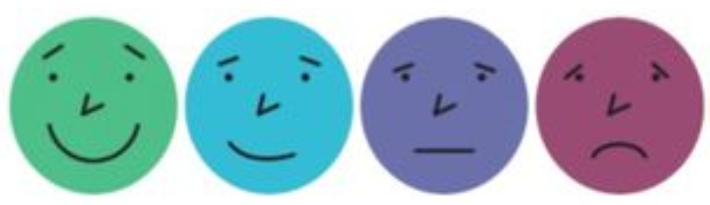

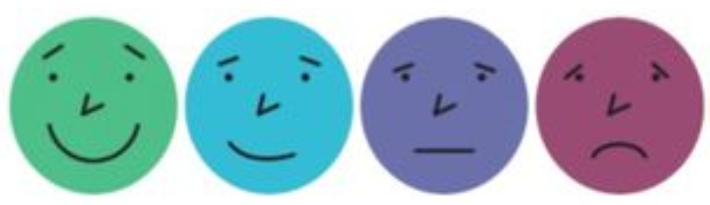

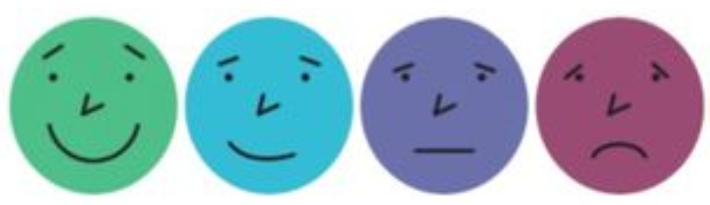

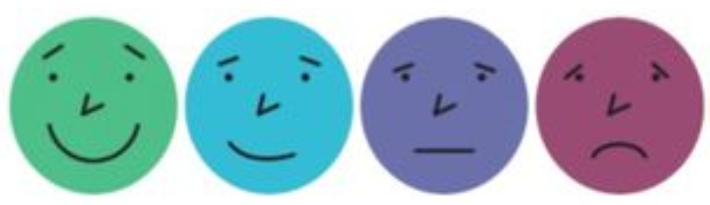


Very bad Bad Good Very Good
