# Supplementary material for: Home-Based mHealth Platform (Active-Feet) for Children With Idiopathic Toe Walking: Design, Development, and Acceptability Study
Source: JMIR Rehabil Assist Technol. 2025 Aug 26;12:e60867. doi: 10.2196/60867 (PMC12380407; doi:10.2196/60867)
Supplement: Multimedia Appendix 4 [file rehab-v12-e60867-s004.docx]

|  | Median | 25% IQR | 75% IQR |
| --- | --- | --- | --- |
| Age | **10** | 9 | 11 |
| Weight (kg) PRE | **42,8** | 35,6 | 55,7 |
| Height (cm) PRE | **148** | 138 | 160 |
| Plantar Flexion Right (º) PRE | **70** | 60 | 75,5 |
| Plantar Flexion Left (º) PRE | **70** | 60 | 74 |
| Dorsiflexion with Knee Extended Right (º) PRE | **0** | -3,5 | 0 |
| Dorsiflexion with Knee Extended Left (º) PRE | **1** | -5 | 4 |
| Dorsiflexion with Knee Flexed Right (º) PRE | **10** | 6 | 12 |
| Dorsiflexion with Knee Flexed Left (º) PRE | **10** | 8,5 | 13,5 |
| Knee Extension Right (º) PRE | **0** | -7,25 | 0 |
| Knee Extension Left (º) PRE | **0** | -4 | 0 |
| Popliteal Angle Right (º) PRE | **48** | 40,5 | 55,8 |
| Popliteal Angle Left (º) PRE | **45,5** | 40 | 54 |
| Hip Flexion with Knee Extended Right (º) PRE | **61** | 51,5 | 77,3 |
| Hip Flexion with Knee Extended Left (º) PRE | **68** | 52 | 72 |
